# Genome-wide association studies for the identification of cattle susceptible and resilient to paratuberculosis

**DOI:** 10.3389/fvets.2022.935133

**Published:** 2022-09-12

**Authors:** Marta Alonso-Hearn, Gerard Badia-Bringué, Maria Canive

**Affiliations:** Department of Animal Health, NEIKER- Basque Institute for Agricultural Research and Development, Basque Research and Technology Alliance (BRTA), Derio, Spain

**Keywords:** paratuberculosis, genome-wide association study, disease tolerance, susceptibility, animal breeding, resistance

## Abstract

*Mycobacterium avium* subsp. *paratuberculosis* (MAP) causes Johne's disease or paratuberculosis (PTB), with important animal health and economic implications. There are no therapeutic strategies to control this disease, and vaccination with inactivated vaccines is limited in many countries because it can interfere with the intradermal test used for bovine tuberculosis detection. Thus, infected animals either get culled after a positive ELISA or fecal PCR result or die due to clinical disease. In this study, we review recent studies aimed to discover genetic markers which could help to identify and select cattle less susceptible and more resilient to PTB. In recent years, the genotyping and subsequent imputation to whole-genome sequence (WGS) has allowed the identification of single-nucleotide polymorphisms (SNPs), quantitative trait loci (QTL), and candidate genes in the *Bos taurus* genome associated with susceptibility to MAP infection. In most of these genome-wide association studies (GWAS), phenotypes were based on ante-mortem test results including serum ELISA, milk ELISA, and detection of MAP by fecal PCR and bacteriological culture. Cattle infected with MAP display lesions with distinct severity but the associations between host genetics and PTB-associated pathology had not been explored until very recently. On the contrary, the understanding of the mechanisms and genetic loci influencing pathogen resistance, and disease tolerance in asymptomatic individuals is currently very limited. The identification of long-time asymptomatic cattle that is able to resist the infection and/or tolerate the disease without having their health and milk production compromised is important for disease control and breeding purposes.

## Economic and social impact of bovine paratuberculosis

*Mycobacterium avium* subsp. *paratuberculosis* (MAP) infections affecting domestic and wild ruminants represent a global major issue on animal health, recognized by the World Organization for Animal Health (OIE) (1), which requires member countries to maintain epidemiological surveillance with notification of disease cases. Clinical signs of MAP infection in cattle include diarrhea, progressive loss of body condition in the animals, and eventually their death (2). Paratuberculosis (PTB) is responsible for significant economic losses in dairy herds worldwide due to decreased milk production, increased management costs, and premature culling or death from clinical disease (3–5). More than 50% of dairy cattle herds are positive for MAP antibodies in the USA and Europe and, therefore, the disease is endemic in these areas (6, 7). In Canada, the economic losses caused by PTB were estimated at $50 CAN per cow per year in MAP-infected herds (8). In Ireland, a profit margin reduction between €168 and €253 was estimated for a cow on a PTB-affected farm (9). The economic impact of PTB on the US dairy industry has been estimated between US $250 M per year to US $1.5 billion annually, with a net return of almost US $100 less per cow in a positive herd than in a negative herd (10). The economic impact of PTB in Europe has been estimated at US $364.31 million per year (11). There is no cure for PTB, and MAP control across the globe has proven to be difficult. MAP survives pasteurization and could enter the human food chain through meat, dairy products, and untreated water supplies (12). It has been detected in samples of patients with Crohn's disease (CD), ulcerative colitis, and idiopathic inflammatory bowel disease (IBD)-associated colorectal cancer (13–15). MAP has also been postulated as a possible trigger in several human autoimmune diseases such as rheumatoid arthritis, multiple sclerosis, and type I diabetes (16–19).

## Factors affecting PTB control

Many control programs for PTB based on vaccination or testing and culling of test-positive cows have been developed worldwide. Commercial inactivated vaccines against bovine PTB are very successful in reducing MAP presence in feces and tissues and in increasing both milk production and the productive life of cattle (20, 21). However, PTB vaccination is not allowed in most European countries due to its interference with *Mycobacterium bovis* detection tests (22). PTB control is currently based on testing and culling and avoiding MAP transmission to susceptible animals by enhancing on-farm biosecurity measures (23, 24). Some factors that hamper the success of such control programs include the lack of compliance with management protocols, the use of tests with low sensitivities to identify all the infected cattle, and the purchase of infected replacement animals which cause new introductions (25). MAP infection occurs primarily through the fecal-oral route, and clinical onset takes place around calving when animals are 18 months or older. According to their extension in the intestine, cellular infiltrate, and amount of MAP, PTB-associated lesions were classified into focal, multifocal, and diffuse (diffuse paucibacillary or lymphoplasmacytic, diffuse intermediate, and diffuse multibacillary or histiocytic) (26, 27). Focal lesions consist of small granulomas in the ileal and jejunal lymph nodes or the ileocecal lymphoid tissue. Multifocal lesions are middle-size granulomas that appear in the apex of some intestinal villi and are formed by groups of macrophages, surrounded by lymphocytes. The focal and multifocal lesions do not cause diffuse enteritis or modify the normal architecture of the intestine. In contrast, the diffuse lesions are associated with a diffuse infiltrate and severe enteritis affecting different intestinal locations and lymph nodes. High bacterial burden, clinical signs, and gross lesions are mainly associated with the presence of diffuse multibacillary lesions.

## Disease tolerance and susceptibility and resistance to the infection

**Figure 1:**
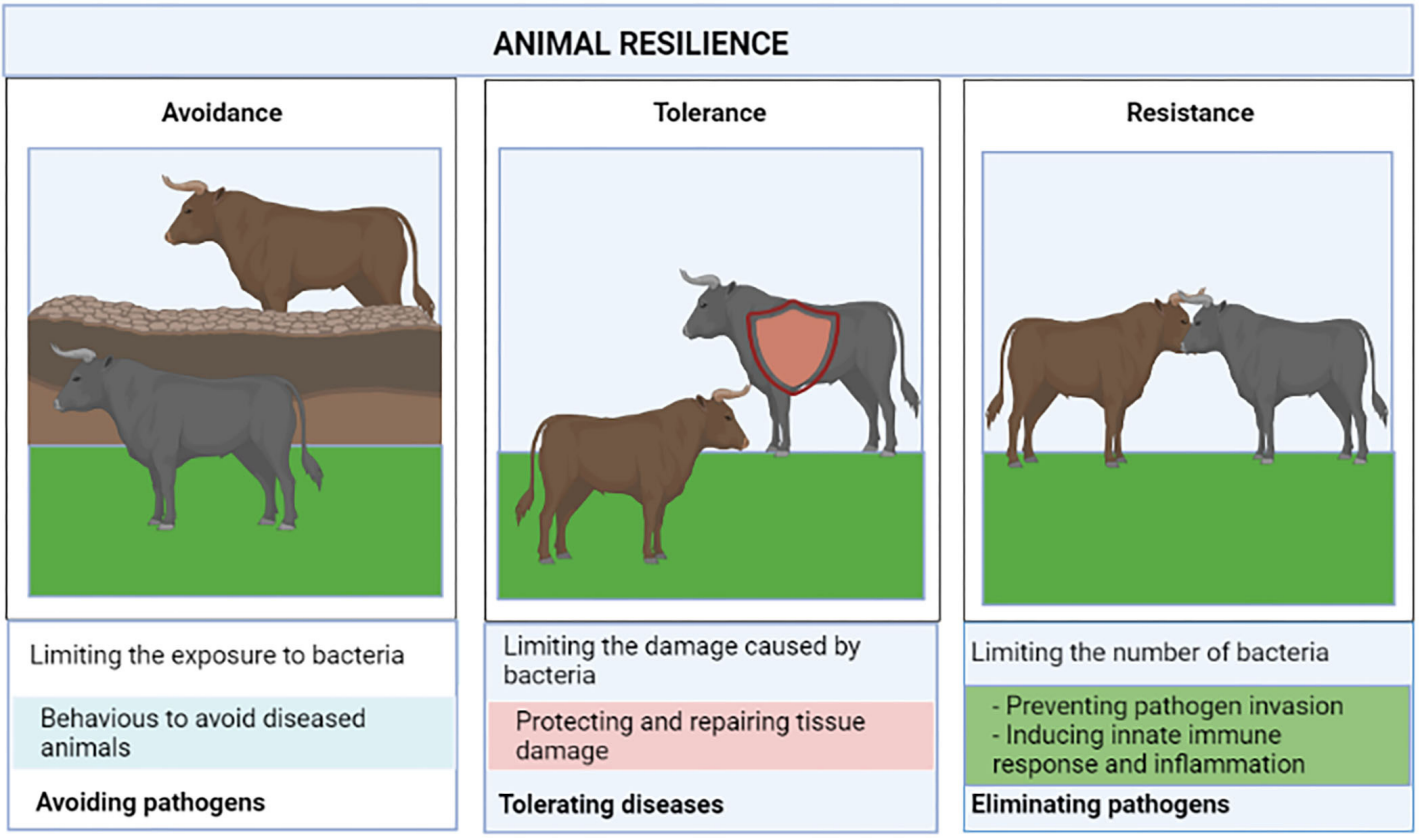
Resilient animals defend themselves against pathogens using the complimentary strategies of avoidance, disease tolerance, and pathogen resistance.

The response to MAP infection is complex and heritable which leads to differences between individuals. Some animals are susceptible to the infection and develop clinical signs while others are resilient and long-term asymptomatic animals. Host defense strategies against infectious diseases are comprised of pathogen avoidance, disease tolerance, and resistance to the infection (Figure 1). Pathogen avoidance refers to the behaviors that animals use to avoid infection caused by pathogens. Resistance is defined as “the ability of the host to prevent invasion (i.e., absence of a target receptor) or to clear the pathogen at the early stage of infection by mounting a protective innate immune response” (28). Disease tolerance is defined as “the mechanisms that decrease host susceptibility to tissue damage, or other fitness costs caused by pathogens or by the immune response” (29). Unlike resistance, disease tolerance does not necessarily imply changes in the pathogen load. Therefore, three major outcomes following exposure to MAP infection can be established: (i) Susceptible host: individuals who progress to clinical disease, (ii) Resistant host: Individuals who can prevent bacterial entry or eliminate the bacteria by inducing innate immune responses at the early stage of the infection, (iii) Tolerant host: If innate immunity is unable to eliminate MAP, the host might initiate disease tolerance mechanisms to prevent and repair tissue damage. The contribution of host genetics is one of the fundamental issues in understanding disease tolerance and susceptibility and resistance to MAP infection.

## Host genetics is associated with susceptibility to MAP infection

The application of animal genetics in breeding programs is currently an important motor for efficient livestock production, not only to increase performance and productivity but also to ensure the resilience and health of livestock while improving their longevity. A widely accepted strategy to reduce or eliminate animal diseases is through the selective breeding of animals with reduced susceptibility and/or enhanced resilience against specific pathogens. Over the last decade, specific candidate genes provided evidence for the existence of single nucleotide polymorphisms (SNPs) associated with differences in susceptibility to bovine PTB (30). The identification of SNPs, quantitative trait loci (QTLs), and candidate genes associated with PTB susceptibility is extremely important not just for breeding strategies but also to understand the mechanisms involved in the pathogenesis of the disease. Previous studies established that the heritability (*h*^2^) estimates of susceptibility to MAP in Friesian cattle ranges from 0.03 to 0.27 (31, 32). The genotyping of SNPs with chips of low (LD), medium (MD), and high density (HD) and subsequent imputation to whole-genome sequences (WGS) have allowed the identification of QTLs and candidate genes in the *Bos taurus* genome associated with susceptibility to MAP infection. Table 1 shows recent studies designated to identify SNPs associated with susceptibility to MAP infection, and Supplementary Table 1 summarizes the candidate genes identified in these studies. Although the cellular immune response is considered the protective effector arm of the immune system for intracellular infections including MAP infection, there have not been GWAS studies published with hereditability estimates based on skin tests or IFN gamma. In most of these GWAS, phenotypes were based on ante-mortem test results such as serum ELISA, milk ELISA, and MAP detection by culture or PCR from fecal samples. We recently identified SNPs, QTLs, and candidate genes associated with ante-mortem (serum ELISA) and post-mortem (tissue PCR and culture) diagnostic definitions in a common set of Spanish Holstein cattle (*N* = 983) using WGS data (46). We observed that the combination of diagnostic tests increased the *h*^2^ estimates, with the highest *h*^2^ obtained for the combination of ELISA-tissue PCR-tissue culture (*h*^2^ = 0.139).

**Table 1:** Genome-wide association studies designed to identify SNPs associated with susceptibility to MAP infection.

**Phenotype**	**Genotype**	**Chromosomes (SNPs number)**	**References**
Tissue culture	MD	BTA1 (3), BTA3 (1), BTA5 (2),1 BTA7 (1), BTA8 (1) BTA9 (4), BTA16 (2), BTA21 (1), BTA23 (1)	(33)
ELISA, fecal culture	MD	BTA2 (2), BTA3 (1), BTA4 (2), BTA5 (1), BTA6 (2), BTA7 (2), BTA9 (2), BTA10 (1), BTA13 (5), BTA14 (1), BTA15 (2), BTA16 (4), BTA17 (3), BTA18 (1), BTA20 (4), BTA21 (4), BTA22 (1), BTA23 (2), BTA25 (2), BTA26 (5), BTA29 (3)	(34)
ELISA	MD	BTA8 (1), BTA9(1), BTA11 (1), BTA12 (5), BTA27 (1)	(35)
ELISA	MD	BTA1 (1), BTA5 (7), BTA6 (2), BTA7 (5), BTA10 (4) BTA11(1), BTA14 (2)	(36)
ELISA, tissue culture	MD	BTA1 (4), BTA6 (1), BTA7 (1), BTA12 (3), BTA13 (1), BTA15 (1), BTA16 (1), BTA21 (1), BTA22 (1), BTA23 (1), BTA25 (1)	(37)
Milk ELISA	MD	BTA4 (1), BTA15 (5), BTA18 (1), BTA28 (5)	(38)
ELISA, fecal culture	MD	BTA2 (38), BTA6 (10), BTA7 (9), BTA8 (8), BTA15 (14) BTA17(18), BTA29 (16)	(39)
ELISA, Fecal culture	LD	BTA1 (3), BTA3 (7), BTA5 (1), BTA6 (2), BTA7 (1), BTA10(1), BTA11 (2), BTA13 (3), BTA16 (1), BTA17 (2), BTA19 (1), BTA23 (7)	(40)
Tissue culture	WGS	BTA3 (1), BTA8 (2), BTA10 (1), BTA12 (2), BTA14 (1), BTA16 (4), BTA21 (1), BTA22 (8)	(41)
Milk ELISA	HD	BTA1 (384), BTA7 (9), BTA9 (16), BTA10 (2), BTA14 (3) BTA15 (5), BTA17 (29), BTA19 (63), BTA21 (2), BTA25 (11), BTA27 (10)	(42)
ELISA	HD	BTA1 (1), BTA2 (2), BTA3 (1), BTA6 (1), BTA7 (5), BTA8 (1), BTA11 (2), BTA13 (2), BTA16 (1), BTA18 (2), BTA20 (1), BTA22 (1), BTA23 (4), BTA24 (1), BTA27 (1)	(43)
ELISA	HD	BTA5 (3), BTA6 (1), BTA7 (3), BTA10 (8), BTA14 (3), BTA15 (3), BTA16 (17), BTA20 (2), BTA21 (1)	(44)
ELISA	WGS	BTA1 (9), BTA3 (2), BTA5 (1), BTA6 (1), BTA8 (1), BTA9 (5), BTA10 (1), BTA11 (3), BTA13 (3), BTA14 (2), BTA18 (2), BTA21 (1), BTA23 (4), BTA25 (1), BTA26 (1), BTA27 (1), BTA29 (2)	(45)
ELISA, tissue culture, tissue PCR	WGS	BTA4 (103), BTA5 (35), BTA11 (43), BTA12 (1), BTA14 (1), BTA23 (126), BTA24 (1), BTA28 (2)	(46)
Histopathology	WGS	BTA1 (43), BTA3 (12), BTA5 (37), BTA7 (37), BTA8 (2), BTA11 (14), BTA13 (6), BTA22 (34), BTA23 (3), BTA24 (31)	(47)
Milk ELISA, ELISA, fecal culture, fecal PCR	WGS	BTA4 (1), BTA7 (4), BTA9 (1), BTA10 (1), BTA12 (1), BTA15 (1), BTA17 (1), BTA18 (3), BTA23 (3), BTA28 (1)	(48)

*LD, low density; MD, medium density; HD, high density BeadChips; and WGS, imputed whole-genome sequences*.

**Figure 2:**
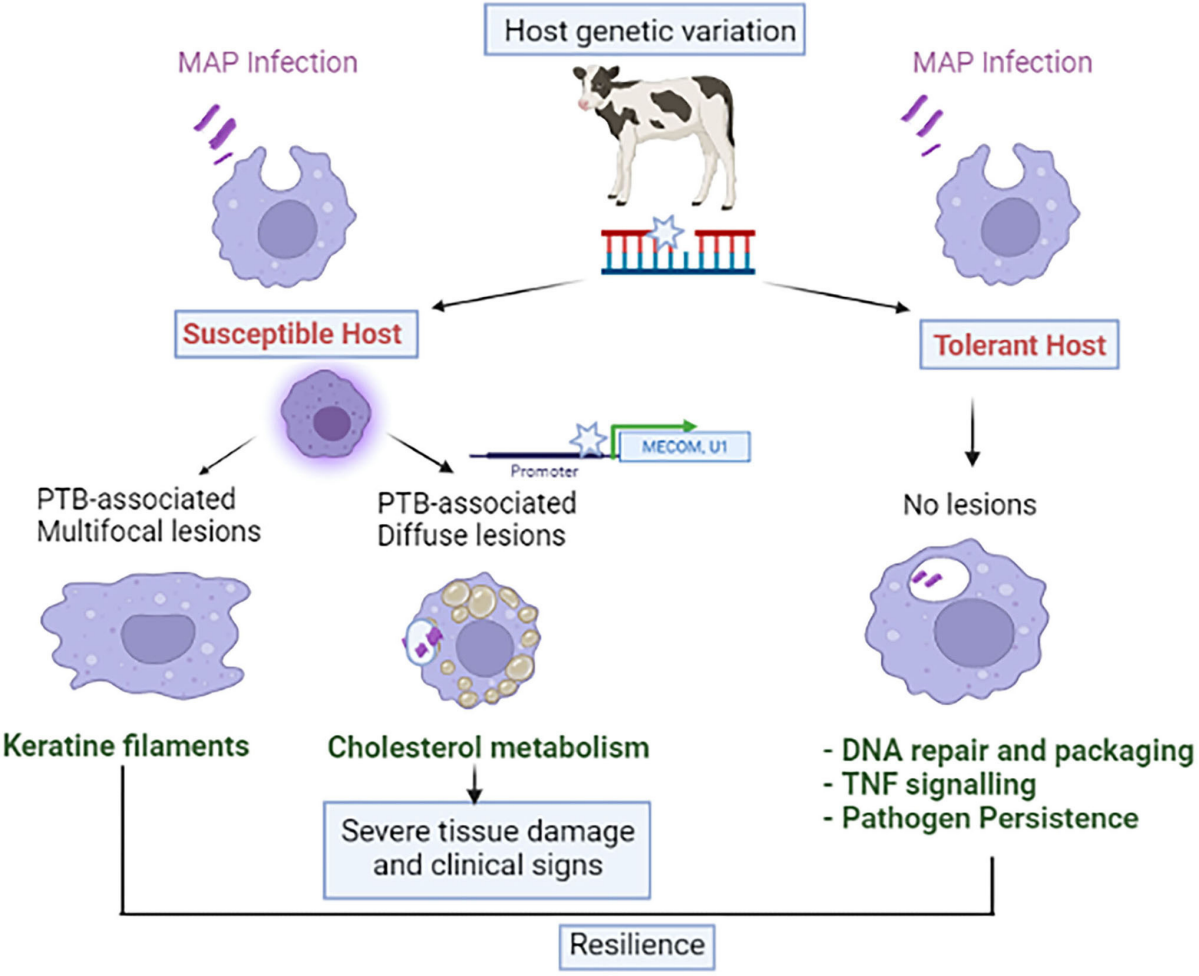
Interaction between host genetics and MAP infection with disease phenotype.

We have recently demonstrated that the post-mortem examination of gut tissues and regional lymph nodes improves the accuracy of the classification of naturally infected animals and provides higher *h*^2^ estimates (47). A total of 192 and 92 SNPs defining 13 and 9 distinct QTLs were associated (*P* ≤ 5 × 10^−7^) with the multifocal (*h*^2^ = 0.075) and the diffuse (*h*^2^ = 0.189) lesions, respectively. No overlap was seen in the SNPs associated with each type of lesion which suggested that distinct genetic variants might control the multifocal and diffuse lesions and that these lesions represent divergent disease outcomes. Pathway analysis with the candidate genes overlapping the identified QTLs revealed a significant enrichment of the keratinization pathway and cholesterol metabolism in the animals with multifocal and diffuse lesions, respectively. The keratin family (KRT), the major subgroup among the intermediate filament family of cytoskeletal proteins, is responsible for maintaining the integrity of the gastrointestinal epithelium, providing resilience against many agents, and regulating various cellular functions such as cellular proliferation, and inflammatory and immune responses. In recent years, it has been demonstrated that the presence of epithelioid granulomas with multifocal distribution in leprosy controls *M. leprae* replication and its dissemination (49). Similarly, we hypothesized that the PTB-associated multifocal granulomas might prevent MAP dissemination and limit tissue damage, representing a signature of disease tolerance. The KRTs might be playing an important role in controlling tissue resilience and bacterial dissemination in animals with this specific type of lesion which might even recover from infection. In contrast, the diffuse lesions represent a disseminated form of the disease characterized by a diffuse inflammatory infiltrate composed mainly of foamy macrophages loaded with cholesterol and large numbers of MAP (Figure 2).

Although GWAS have allowed the identification of SNPs associated with the susceptibility to MAP infection, the genes through which these variants exert their effects are unknown, and only a few functional mutations for PTB have been identified (50, 51). Expression quantitative trait loci (eQTLs) are genetic variants located in gene regulatory regions that alter gene expression. eQTLs can be considered as functional links between genomic variants, gene expression, and ultimately phenotype. Recently, the integration of gene expression data (RNA-Seq) and genotypes (54,609 SNPs per animal) from a cohort of cows naturally exposed to MAP has allowed the identification of 192 and 48 cis-eQTLs associated with the expression of 145 and 43 genes in peripheral blood (PB) and ileocecal valve (ICV), respectively (52). Three of these cis-eQTLs regulating the expression of the *MECOM, eEF1A2*, and *U1* spliceosomal RNA expression were significantly associated with PTB susceptibility (Figure 2). This study identified the first cis-eQTLs involved in gene transcription regulation and PTB susceptibility by integrating genotypes and RNA-seq data.

## Host genetics is associated with resistance to MAP infection

Although we identified a total of 380 SNPs associated (FDR ≤ 0.05; *P* < 5 × 10^−7^) with PTB susceptibility using ante-mortem and post-mortem definitions in a common set of Spanish Holstein cattle (*N* = 983) (46, 47), no conclusions could be drawn regarding host resistance. While test-positive cows are very likely infected, animals could test negative due to the lack of sensitivity of the diagnostic methods or lack of exposure to MAP. In other words, a negative test does not always reflect resistance, which precludes the identification of loci associated with resistance. Few studies have attempted to identify genetic variants associated with resistance to MAP infection in cattle. In 2010, Pant et al. (53) genotyped 232 Canadian dairy Holstein cattle with MAP infection status assessed by ELISA and milk ELISA. Using this approach, eleven SNPs on BTA1, 5, 6, 7, 10, 11, and 14 were associated with resistance to MAP infection. However, the population size was small (232 animals) and the classification of the animals based only on ELISA test results could lead to misclassification due to the lack of sensitivity of the ELISA. More recently, Sanchez et al. (54) carried out a multi-breeding GWAS study of 1,644 Holstein Friesian and 649 Normande cows with imputed WGS. In this study, control cows without clinical signs were tested by serum ELISA and fecal PCR at least four times. Clinical cases were confirmed with both ELISA and PCR tests. Thus, animals were classified as controls, cases without clinical signs, and cases with clinical signs. With this approach, a total of 2,827 resistance-associated SNPs distributed in 20 quantitative trait loci (QTLs) were identified. However, the threshold used to determine the significance of the identified variants was very low (*P* < 10^−6^), and the multiple positive correction was limited only to 50,000 independent tests.

## Host genetics is associated with PTB tolerance

Regarding disease tolerance, genetic variants that confer greater fitness displaying positive or advantageous selection can potentially be observed in the host genome (55). Recently, we have searched for genetic loci associated with tolerance to PTB by using WGS data from infected Spanish Holstein cows with MAP detected by tissue PCR and bacteriological culture but without lesions in gut tissues and regional lymph nodes. A PTB tolerant animal was PCR and culture-positive (infected) but no lesions could be observed in the histopathological analysis of gut tissues (no disease) (56). Although an earlier paper described a GWAS for PTB tolerance (57) with a subsequent refinement of genetic regions associated with MAP tissue infection and tolerance to PTB (58), our GWAS is the first to complete an analysis of the genetic markers associated with tolerance to PTB using WGS data and epidemiological data based on three diagnostic tests; histopathology, tissue PCR, and bacteriological culture. Results from our study do not corroborate those of Zanella et al. (57, 58), who identified a tolerance-associated region of 6.5 Kb on BTA15 which we did not identify. In the studies by Zanella et al., tolerance measured the relationship between MAP infection intensity (level of MAP in gut tissues) and fitness (level of MAP fecal shedding). The amount of MAP in feces was used in this study as a proxy for fitness but to define tolerance, direct disease outcomes measurements are critical. In our study, PTB-tolerant cows are infected animals with positive PCR and bacteriological culture results but without lesions in gut tissues. Our results suggest that there is genetic variation associated with PTB tolerance (*h*^2^ = 0.55) and that this variation is indicative of an immunogenic profile in the PTB tolerant animals designed to control bacterial growth, modulate inflammation, and limit tissue damage (Figure 2) (56). Some of the identified QTLs overlapped with QTLs previously associated with PTB, bovine tuberculosis, mastitis, somatic cell score, bovine diarrhea virus persistent infection, tick resistance, and length of productive life. However, the need to slaughter animals to measure MAP load in tissues as an indicator of tolerance limits its employment. Thus, novel tools to measure PTB tolerance must be developed.

## Conclusion

Since it is well recognized that not all asymptomatic animals will progress into clinical cases during their productive life, the identification of genetic markers associated with PTB tolerance might help farmers or animal health managers to select which infected cows should not be culled which in turn should increase the benefit/cost of their control program. In addition, the introduction of novel genetic variants associated with PTB susceptibility and tolerance into marker-assisted breeding programs would help producers to select less susceptible cattle, and more tolerant to PTB and likely to other bovine diseases as well, ultimately preventing economic losses and reducing antimicrobial use. Preventing endemic and chronic diseases such as PTB by selecting resilient cattle is important for sustainable and efficient dairy farming and the maintenance of the rural economy. Although this is a long-term control strategy, the benefits of breeding resilient animals could be permanent and transferred to subsequent generations.

## Author contributions

MAH conceived and coordinated the project and wrote the manuscript. All authors contributed to the article and approved the submitted version.

## Funding

Financial support for this study was provided by a grant (RTI2018-094192-R-C21) funded by MCIN/AEI/10.13039/501100011033 and by FEDER, Una manera de hacer Europa. MC and GBB have been awarded fellowships from MCIN/AEI/10.13039/501100011033 and FSE Invierte en tu futuro; grants FPI2016-00041 and PRE2019-090562, respectively.

## Conflict of interest

The authors declare that the research was conducted in the absence of any commercial or financial relationships that could be construed as a potential conflict of interest.

## Publisher's note

All claims expressed in this article are solely those of the authors and do not necessarily represent those of their affiliated organizations, or those of the publisher, the editors and the reviewers. Any product that may be evaluated in this article, or claim that may be made by its manufacturer, is not guaranteed or endorsed by the publisher.
